# Tumor suppressor proteins restricts LINE-1 retrotransposition

**DOI:** 10.1186/1742-4690-10-S1-P43

**Published:** 2013-09-19

**Authors:** Misao Kuroki, Mariko Yasuda-lnoue, Shiori Nakashima, Yasuo Ariumi

**Affiliations:** 1 Center for AIDS Research, Kumamoto University, Kumamoto, Japan

## Background

Long interspersed element 1 (LINE-1) is a retroelement comprising about 17% of the human genome, encoding ORF1 with an RNA interaction domain and ORF2 with endonuclease and reverse transcriptase activities. Similar to retroviruses, LINE-1 replicates in the host via an RNA intermediate that is reverse transcribed and integrated into the host genome. LINE-1 retrotransposition has resulted in genetic diseases. In fact, LINE-1 insertion was recently found in several human tumors. However, the life cycle of LINE-1 is not fully understood. Therefore, we examined identification and characterization of host factors affected the retrotransposition of LINE-1.

## Materials and methods

We used the pL1_RP_-EGFP plasmid, which contains an enhanced green fluorescent protein (EGFP)-based retrotransposition detector cassette. The retrotransposition rate of LINE-1 in 293T cells was determined by flow cytometry after the cotransfection of pL1_RP_-EGFP with plasmids expressing DEAD-box RNA helicases (DDX1, DDX3, DDX5, DDX6, DDX17, DDX21, and DDX56), cancer related proteins (p53, p21, Pin1 and PML isoforms I-VI) or P-body components (MOV10, Ago2, APOBEC3F, and APOBEC3G). We observed the subcellular localizations of LINE-1 ORF1 protein and host factors by using confocal laser scanning microscopy. We also examined immunoprecipitation to analyzed the interaction of LINE-1 ORF1 with the host factor(s).

## Results

We found that MOV10 markedly inhibited the retrotransposition of LINE-1 as well as APOBEC3G/F. Accordingly, LINE-1 ORF1 colocalized with MOV10 or APOBEC3G in P-bodies. In addition, LINE-1 ORF1 was also found in stress granules induced after the treatment with 0.5mM NaAsO_2_ for 30 minutes, indicating that LINE-1 ORF1 is both P-body and stress granule component. Furthermore, immunoprecipitation analysis showed that MOV10 and APOBEC3G bound to LINE-1 ORF1, suggesting the inhibitory mechanism. Moreover, we noticed that DDX3, PML IV, p53, p21, Pin1, and Ago2 significantly inhibited the retrotransposition of LINE-1.

## Conclusion

We identified several host factors, including MOV10, DDX3, PML IV, p53, p21, Pin1, and Ago2 as the restriction factors of LINE-1. Thus, several tumor suppressor proteins seem to restrict LINE-1 retrotransposition.

